# *Physalis peruviana* L. (Solanaceae) Is Not a Host of *Ceratitis* *capitata* (Diptera: Tephritidae): Evidence from Multi-Year Field and Laboratory Studies in Colombia

**DOI:** 10.3390/insects10120434

**Published:** 2019-12-04

**Authors:** Martín Aluja, Larissa Guillén, Ángela Castro, Martha Liliana Cárdenas, Maribel Hurtado, Óscar Durán, Emilio Arévalo-Peñaranda

**Affiliations:** 1 Instituto de Ecología, A.C. (INECOL), Clúster Científico y Tecnológico BioMimic®, Carretera Antigua a Coatepec 351, El Haya, Xalapa 91073, Veracruz, Mexico; 2 Instituto Colombiano Agropecuario (ICA), Av. Calle 26 No. 85B-09, Bogotá 110931, Colombia; angela.castro@ica.gov.co (Á.C.); martha.cardenas@ica.gov.co (M.L.C.); maribelhurtado.s@gmail.com (M.H.); oscar.duran@ica.gov.co (Ó.D.); emilio.arevalo@ica.gov.co (E.A.-P.); 3 Agencia de Desarrollo Rural (ADR), Calle 43 # 57-41 CAN, Bogotá 111321, Colombia

**Keywords:** host status, non-natural host, non-conditional host, Uchuva—*Physalisperuviana*, Solanaceae, Medfly—*Ceratitiscapitata*, Diptera: Tephritidae, international trade

## Abstract

Scientifically-based, tephritid fly host status determination lies at the heart of strategic regulatory decisions impinging on international fruit trade. Here we conducted intensive field and laboratory studies with peaches as controls, to determine the host status of *Physalis* *peruviana* for the Medfly—*Ceratitis* *capitata*, as this fruit is experiencing a consumption boom worldwide. A total of 98,132 Uchuvas (local name), collected in Colombia from the plant or the ground over a three-year period (2016–2018) did not yield a single *C*. *capitata* larva or pupa, thus reaching a Probit 9 level with 99.9968% efficacy and 96% confidence level. Field-cage studies with enclosed fruit-bearing Uchuva plants, exposing fruit with an intact, damaged or totally removed husk to the attack of *C*. *capitata*, also failed to yield infestations. Highly artificial choice experiments, exposing gravid females to unripe and fully ripe fruit, resulted in an absence of infestations, even when overripe Uchuvas were artificially damaged. The husk and surface resins/waxes inhibit fly landings on fruit and oviposition activity. Considering our results and the fact that the foliage, husk and fruit of *P*. *peruviana* are repellent/toxic to insects, we conclude that this plant should be treated as a non-natural and non-conditional host of *C*. *capitata*.

## 1. Introduction

Fruit fly (Diptera: Tephritidae) host status determination remains a contentious issue generating disputes between countries over import/export requirements [1,2,3]. In the late 2000s, Aluja and Mangan [4] published an extensive review on the issue, proposing a simple flow chart to facilitate host plant determination worldwide and reduce ambiguity in the terminology commonly used, as inaccurate terms were too engrained in the specialized literature and caused unnecessary and often sterile discussions. In sum, they proposed two basic categories: natural vs. non-natural host and within the latter, nonhost and conditional (potential/artificial) host. Subsequent regulatory guidelines [5] have adopted this fundamental approach as it greatly simplifies host status determination.

**Figure 1:**
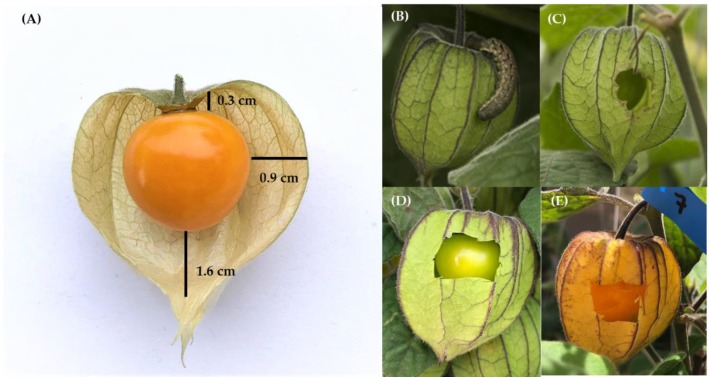
(**A**) Uchuva with calyx partially removed to show the ripe, shiny fruit inside (totally covered by a resin/wax) and illustrate the mean distance (N = 15) between the calyx and the fruit when it is fully ripe; (**B**) noctuid larva feeding on calyx of unripe *P*. *peruviana* fruit; (**C**) hole produced by larvae to reach fruit protected by calyx. This phenomenon lead us to include various treatments in our experimental design to mimic the holes potentially allowing *C*. *capitata* females to reach the otherwise protected fruit (details in Section 2.2.1); (**D,E**) unripe and ripe Uchuvas into which an artificial hole was cut to mimic condition depicted under (**B**).

*Physalis peruviana* L. (Solanaceae), commonly known as “Cape Gooseberry” (South Africa), “Goldenberry” (USA), “Uchuva” (Colombia), “Uvilla” (Ecuador) or “Aguaymanto” (Perú), is an edible fruit that has been known since the Inca empire [6,7,8]. It is indigenous to South America where its original distribution purportedly ranged from Venezuela to Bolivia, including Colombia, Ecuador, Chile and Peru [6,9,10], but is now found in many countries worldwide [11,12] and is sometimes considered a weed [13,14]. In its native range, *P*. *peruviana* is found at altitudes between 800 and 3000 m [15,16] and grows as a bush in forest edges, roadsides and abandoned fields. A fully-grown *P*. *peruviana* plant can reach 1.8 m in height and bear over 300 fruit per plant [10]. The fruit, a berry, once fully developed measures 1.25–2 cm wide, can weigh up to 11 g (typically between 3.5 and 8.5 g) and attains a yellow/orange color when fully ripe [17,18]. It is protected by a thin but sturdy husk (calyx in botanical terms), known in Colombia as “Capacho”, that when fully developed completely covers the fruit until it ripens. This structure can more than double the size of a ripe berry [10]. The fruit is only in contact with the husk at the base (Figure 1A). In the field, the husk is eaten by larvae of Lepidoptera, including various species of Noctuidae, such as *Heliothis* spp. [19], and the flea beetle *Epitrix cucumeris* Harris (Chrysomelidae) [20]. These insects produce holes through which the fruit growing inside the calyx can be seen (Figure 1B,C), a fact we incorporated into our experimental design (Figure 1D,E).

The status of *P*. *peruviana* as a natural or conditional host of fruit flies is contentious. The only two studies claiming purported field infestations by the Medfly (*Ceratitis capitata* (Wiedemann)) and the oriental fruit fly (*Bactrocera dorsalis* (Hendel)) [21,22], are based on historical compilations of data collected by many people over many years, most likely using different fruit collection and handling methods. For example, after conducting a survey spanning eight years (1958–1966), Nakagawa et al. [21] reported one *B*. *dorsalis* and two *C*. *capitata* pupae in 200 *P. peruviana* fruit. In comparison, the same authors reported 5627 pupae reared from 5442 *Malpighia punicifolia* L. fruit, from which 2940 and 18 adults of *B*. *dorsalis* and *C*. *capitata* emerged, respectively. In the case of Liquido et al. [22], their survey spanned 37 years, from 1949 to 1985. These authors reported that one of five *P*. *peruviana* samples, totaling 162 fruit (details on collection methods, fruit handling or year when collections were performed were not provided), contained 11 Medfly larvae/kg of fruit. Unfortunately, none of these reports indicate if the fruit was collected from the plant or ground, if they were ripe or unripe and most importantly, do not provide information on fruit handling and condition i.e., cultivar or ecotype, degree of ripeness, if the calyx was intact or broken, if the fruit had been damaged by birds or insects or if the fruit had an intact peduncle or had natural splits due to extreme ripeness.

In a subsequent survey by Liquido et al. [23], directed at *Bactrocera latifrons* (Hendel) and conducted on the Islands of Hawaii and Maui, out of 47 fruit species collected, 15 were identified as “suitable host plants” (11 within the Solanaceae and four within the Cucurbitaceae), but in this case, *P*. *peruviana* was not found to be infested by *C*. *capitata*. In 18 collections, totaling 1351 fruit (3.26 kg; which means that the fruits were very small as their mean weight was 2.41 g), in one sample (7.7%), *B*. *latifrons* infested *P*. *peruviana* (mean of 3.4 larvae per 100 fruit), but not a single *C*. *capitata* was recovered. The authors interpreted this result as a “niche overlap” between *B*. *latifrons*, *B*. *dorsalis* and *C*. *capitata*, with the first species out-competing the latter two. More recently, Vargas et al. [24] also working in Hawaii, reported not having found infestations by *C*. *capitata* and *B*. *dorsalis* in *P*. *peruviana* samples even though during the first years of their survey, sampling was restricted to damaged fruit “to maximize chances of finding infested fruit”. In the only other publication, we were able to identify addressing possible fruit fly infestations of Uchuva in the field (Ecuador), no pupae of Medfly, *Anastrepha* spp. or *Rhagoletis* spp. were present [25].

In summary, based on all the available literature, the host status of *P*. *peruviana* for *C*. *capitata* is debatable and needs to be revised as several withanolide compounds in *P*. *peruviana,* such as salpichrolide A, C, G [1,2,3] and B [5], have toxic effects on Medfly larva (up to 95% mortality) and also retard larval development [26]. Similarly, Cirigliano et al. [27] found that withanolide E and 4-β-hydroxywithanolide E caused larval mortality in *C*. *capitata*. Baumann and Meier [28] working with the same compounds, reported that these chemicals were present in the husk and were toxic to herbivores. Furthermore, Ascher et al. [29], Glotter [30] and Veleiro et al. [31], and more recently Franco et al. [32], have also reported insect antifeedant, repellent and direct toxic effects in *P*. *peruviana*. For example, Franco et al. [32], identified two sucrose esters, Peruviose A and Peruviose B in the calyx, which in addition to the plentiful resin/wax covering the fruit, render the leaves, calyx and fruit toxic to many insects. Our aim here was to experimentally determine the host status of *P*. *peruviana* for the Medfly, as this fruit is experiencing a consumption boom worldwide and as a result there are increasing export opportunities in Uchuva-producing countries.

## 2. Materials and Methods

### 2.1. Fruit Sampling in the Field and Adult Medfly Trapping

Between May 5th, 2016 and the end of 2018, a total of 98,132 fully ripe *P*. *peruviana* fruit with complete or damaged husks were collected from both standing plants and the ground. These fruits were transported to a laboratory located in the city of Pamplona, Norte de Santander, Colombia (Figure S1). There, following methods described in Aluja et al. [2], the fruits were placed over vermiculite as a pupating medium and checked every third day for the presence of pupae or larvae. Of the 98,132 Uchuvas, 92,503 (630 kg) were collected from 34 commercial Uchuva plantations of varying sizes (0.25 to 1.0 ha) located in the Province of Pamplona, Norte de Santander, Colombia (Table 1). Of the latter fruit, 90,786 (98.1%), were collected from standing plants and 1717 (1.9%) from the ground. Additionally, 5629 Uchuvas (28.15 kg) were collected from ‘feral’ Uchuva plants growing “wild” in the same region along roads, field edges or in patches with wild vegetation. Of these, 5133 (91.2%), were collected from standing plants and 496 (8.8%) from the ground (Table 1). None of the owners of the commercial plantations where we sampled fruit kept formal/systematic records of the agrochemicals they applied, but based on the types of products used by the owner of the experimental plantation we worked in, the insecticides and fungicides occasionally applied by local Uchuva growers are mild and have short residuality periods (details under Section 2.2.1 Study Sites and Experimental Treatments).

During the same three-year sampling period (2016–2018), we also surveyed fruit of 54 plant species on a weekly basis along roads, commercial plantations and in backyard gardens in the vicinity of Uchuva and peach (*Prunus persica* [L.] Stokes) plantations (Table S1), to ascertain on which plants *C*. *capitata* and some species of *Anastrepha* and *Neosilba* were able to develop. Variable numbers of fruit/kg were collected according to availability (exact information provided in Table S1), transported to the same laboratory in which Uchuva samples were kept, and processed as described above.

Finally, 250 Jackson and 227 McPhail traps were hung in known *C*. *capitata* hosts or other types of trees at 238 field sites to indirectly estimate Medfly population size over time. Details of the trap placement area, including the locations of all sampling and trapping sites, are given in Figure S2.

#### Probit 9 Determination

We ran a Probit 9 analysis using the 98,132 fully ripe *P*. *peruviana* fruit collected in commercial plantations and in the wild, as this calculation is critical in fruit fly host-plant status determination procedures [3].

### 2.2. Field-Cage Studies

#### 2.2.1. Study Sites and Experimental Treatments

Studies were conducted in 2018 during the rainy (June) and dry (November/December) seasons in “Predio Sisará”, located at 2559 MASL in the Municipality of Cácota, Norte de Santander, Colombia at 7°15′29.27” N and 72°38′05.57” W where we worked with Uchuvas, and in “Predio Buenavista”, located at 2077 MASL in Tane, Municipality of Chitagá, Norte de Santander, Colombia at 7°15′05.58” N and 72°34′42.78” W, where we worked with peaches. These sites are separated by a straight-line distance of just 6.19 km. Overall environmental conditions (mean + SE annual values during 2010–2016) in the study area were: 15.5 ± 0.076 °C, 76% ± 0.45% relative humidity (RH) and 548 ± 63.83 mm rainfall that mainly occurs between May and August (Instituto de Hidrología, Meteorología y Estudios Ambientales, Colombia). The exact environmental conditions inside the field enclosures when tests were run in the case of Uchuvas were as follows: June 18.4 ± 0.2 °C (with peaks of up to 25 °C), 57.2% ± 0.6% RH, and 38 mm rainfall; December 20.0 ± 0.47 °C (with peaks of up to 27 °C), 60.03% ± 0.86% RH, and 0 mm rainfall. The Uchuva fruit-bearing plants used in the dry season were water-stressed, as the plantation had no artificial irrigation system (we will consider the relevance of this in the Discussion section). In the case of the peach production site, climatic conditions were as follows: June 2018, 17–24 °C, 76.5% ± 1.56% RH and 68 mm rainfall; December 2018, 16–28 °C, 36%–76% RH; no rainfall occurred during the experimental period, as was the case in the neighboring Uchuva site.

The Uchuva site was selected in the Municipality of Cácota, where there was 134 ha of peaches (*P*. *persica* cvs “Gran Jarillo” and “Jarillo”), a preferred host of *C*. *capitata*. It is common to find Uchuva plantations adjacent or close to peach orchards. In Tane (peach site), 422 ha of peaches are grown. As a result, large populations of Medfly are present year-round in this region. During the study period (2015–2017), the mean annual fly/trap/day index (FTD) value was 1.28 ± 0.038, with peaks of up to 3.5 FTD on week 15 of 2017 in Cácota and 6.4 FTD on week 52 of 2017 in Tane. That is, we selected sites within Colombia that presented ideal conditions for the purposes of the study (i.e., high Medfly populations), with the caveat that at such high altitudes, temperatures are low even during the summer months (rainy season). Cool temperatures reduce fly activity in the early morning and late afternoon.

**Figure 2:**
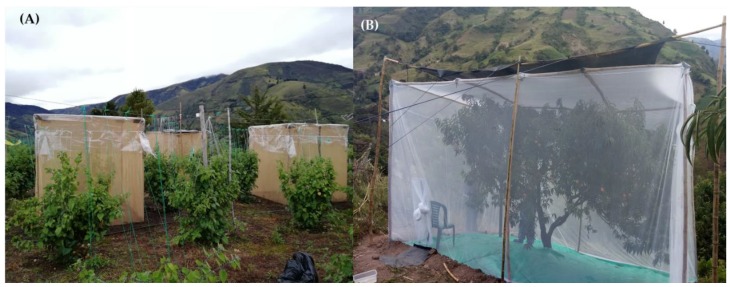
(**A**) Field cages completely covering a single fruit-bearing *P. peruviana* plant of the ‘Colombia’ ecotype and (**B**) *P. persica* (cv. ‘Jarillo’) tree. Observations were conducted simultaneously in the 2018 rainy (June) and dry (late November–December) seasons in “Predio Sisará”, Municipio de Cácota (Uchuva) and at “Predio Buenavista”, Municipio de Chitagá (Peach), both located in Departamento Norte de Santander, Colombia.

In the Uchuva plantation (‘Colombiana’ ecotype) that was rented specifically for this study, there were 1800 plants in an area of 0.75 ha that was not treated with any insecticide or fungicide one month prior and during our experiments. Prior to this, the grower applied in April 2018 Glifosol^®^, (N-(Phosphonomethyl) glycine), Nogueres, France and in October 2018 Exalt™ (Spinetoram, Indianapolis, IN, USA) against leaf miners, Antracol^®^ (Propineb, Monheim, Germany) as a broad-spectrum fungicide, and Vertimec^®^ (Abamectin, Monthey, Switzerland) against aphids and mites. Based on the known residuality of all products, the month free of applications prior to our experiments guaranteed that all test plants were free of residues. We individually covered ten fully-developed *P*. *peruviana* plants located at the center of the experimental site. Four of these plants were studied in each season, plus two additional plants that were caged at the end of November 2018 for preliminary tests and observer-training purposes. The plants were completely healthy and had no signs of fungal or viral disease. Each plant was enclosed in 2 × 2 × 2 m enclosures (20 × 20 mesh; Lumite Inc.^®^, Alto, GA, USA) (Figure 2). Dark greenhouse mesh (high-density polyethylene, 50% transmittance, r 800/m^2^) was placed on the roof of each enclosure to provide shading (details in Aluja et al. [2]). 

**Figure 3:**
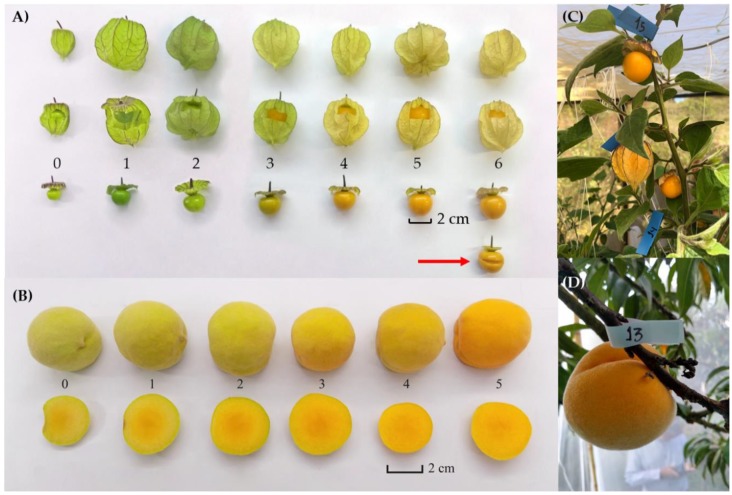
Photographic depiction of all treatments used in field cage and laboratory experiments illustrating Uchuvas and peaches of varying degrees of ripeness (2–6 in case of *P*. *peruviana* and 3–5 in case of peaches). (**A**) Uchuvas with an intact calyx, an artificial hole mimicking damage inflicted by lepidopterous larvae (details in Figure 1) or with calyx removed to allow direct access to fruit by sexually mature *C*. *capitata* females. We performed a lateral incision on a totally ripe fruit (fruit pointed by red arrow) to facilitate oviposition by females in our choice experiment under highly artificial laboratory conditions; (**B**) Peaches of three degrees of ripeness used in experiments (3, 4, 5) showing fully colored pulp (an indication of ripeness); (**C**) Close-up of Uchuva plant illustrating different fruit labeled with small colored tags at the peduncle to distinguish among treatments; (**D**) Close-up of a peach (degree of ripeness five) with an ovipositing *C*. *capitata* female and showing the numbered tag used to identify individual fruit.

In the case of the Uchuva plants used for experimentation during the tests performed in June 2018 (rainy season), three days before we released flies for observations, we removed all floral buds, flowers and fruit of degrees of ripeness zero and one. To facilitate observations, we also removed several branches and leaves. With the remaining fruit of ripeness degrees two–six, we left 75 fruit per cage and removed all the rest. The 75 fruit left on the plant were equally divided into 15 treatments (Figure 3A). To facilitate notations, each type of fruit was labeled with a small colored tag on the peduncle (Figure 3C,D). In the case of the December 2018 (dry season) tests, we performed the same procedure as in June, and waited for 72 h after plants had been pruned before releasing flies into cages. The latter procedure was necessary, as during preliminary tests at the end of November 2018 we detected signs of intoxication in flies, possibly caused by volatiles emitted by the water-stressed and recently pruned *P*. *peruviana* plants (Figure S3). In contrast to our June and December tests, in late November we released flies into the two training cages the same day (late afternoon) after the Uchuva plants had been pruned and observed them the next day. We therefore ran the formal tests in December with four caged Uchuva plants allowing the wounds inflicted on the plants through our pruning procedure to heal and dry before any flies were released (as had been the case in June). The caged peach tree exposed to flies at the end of November in which flies exhibited normal behaviors was used as an additional replicate (details follow).

As the peach trees present in the Uchuva plantation or surrounding it did not bear enough fruit at the time the tests were performed, we worked in a commercial 12 ha peach orchard (“Predio Buenavista”) close to the Uchuva plantation (in a straight-line distance of 6.19 km) where two peach cultivars were grown: “Gran Jarillo” and “Jarillo” (the most susceptible to Medfly attack). We enclosed five of the best-looking peach trees with plentiful fruit in 6 × 5 × 3.3 and 6.5 × 5.5 × 4 m cages (length × width × height) covered with Tulle cloth on all sides and the roof (Figure 2). To provide for adequate shading, we used the same dark greenhouse mesh with which we covered the Uchuva field-cages. The first two trees used during the rainy season (June 2018) were “Gran Jarillo” (ca. 3 m height and with a canopy of ca. 5 m width), and the additional three trees used during the dry season (one at the end of November and two during December 2018) were “Jarillo” (ca. 3.5 m height with a canopy of ca. 5 m width). Each tree had between 1400 and 2400 fruit of varying degrees of ripeness. We selected 75 fruit for our experiment (the same number of fruit as used for the enclosed Uchuva plants), 25 at each stage representing the three degrees of ripeness that are most susceptible to Medfly attack (Figure 3B). Fruits were labeled with a small colored tag in the peduncle as was the case with Uchuvas (Figure 3D). All the remaining fruit were removed from the trees. This orchard was not treated with any insecticide or fungicide prior to testing, or during the experimental period.

#### 2.2.2. Experimental Fly and Fruit Handling

All the flies used in the experiments were wild and originated from infested peaches collected in the vicinity of the study site or areas nearby. For the June 2018 tests, a total of 202 kg of peaches were collected in the Province of Pamplona yielding 6796 pupae and 5156 adults. For the late November and December 2018 tests, 249 kg of peaches were collected in the same province, yielding 10,815 pupae and 9427 adults. Once larvae had pupated, pupae were left in the vermiculite for at least eight days to avoid damaging the developing adult and possibly affecting its behavior. Between 150 and 200 pupae were then transferred to 500 mL plastic containers, which were in turn placed inside 60 × 30 × 30 cm Plexiglas cages covered with Tulle cloths for adult emergence. Previously, 20 pupae were weighed individually as an indicator of the size of the flies used in the experiments. In emergence cages, flies were fed ad libitum with a 3:1 sugar:hydrolyzed protein solution. Water was provided through a moistened cotton pad in an additional container. Once flies reached sexual maturity, three sets of 15 female/male pairs were placed in similarly-sized cages and were exposed to artificial oviposition devices (3 cm diameter agar spheres wrapped in Parafilm) to ascertain fertility, as described by Jácome et al. [33]. Two days prior to release and during assays, we dissected all eggs from the agar spheres to measure the number of enclosed eggs in each cohort, following Jácome et al. [33]. The fact that the flies released into Uchuva and peach cages were in optimal condition and sexually mature was confirmed as a sample of 511 eggs dissected from 24 agar spheres had a fertility of 68.7%. We note that we used the same food and water dispensers placed on Uchuva or peach branches in the field cages to guarantee ad libitum access to food and water to flies during the observation period (details follow).

In both the rainy and dry seasons, we released 225 sexually mature *C. capitata* females and 75 males (total of 300 flies/cage) between 17:00 and 18:00 h in each of the experimental cages one day prior to testing. Thereafter, flies were left in the cages for 72 h (three complete days). Prior to starting observations (the day after fly release), we thoroughly inspected the floor to count dead flies and replaced them with individuals from the same cohort that had been kept overnight in a cage close to the release plants. Once tests and observations were completed, we removed all flies manually (including dead ones on the floor), using an aspirator. Four days after having released the flies, all ripe fruit were harvested and transported to the laboratory. All the remaining fruit were left inside the field cages until fully ripe, then transported to the laboratory and placed individually in a plastic container over a pupation medium. Seven days after harvest, we started to perform daily inspections of all plastic containers in search of pupae. In the case of Uchuva, fruits were left inside the containers for a maximum period of 90 days to allow all potential larvae to fully develop and pupate freely. In the case of peaches, fruits were removed much earlier as they rotted quickly and oozing juices and fungi growing on fruit could harm pupae. Therefore, the pupae were gently transferred to another container with moistened vermiculite daily until adult flies emerged.

#### 2.2.3. Observation Protocol

Detailed, systematic observations on fly behavior inside cages, were only conducted during the three-day exposure period in December 2018 (dry season). One observer per cage (including the two peach trees) observed fly behavior over an eight-hour continuous period from 10:00 to 18:00 h. Observers had to follow a strict clothing protocol, wearing light blue hospital operation room gowns and caps. The use of perfumes and deodorants was prohibited. The observation protocol was as follows: At the beginning of every hour, the observer walked slowly and carefully (without rapid body movements) around the Uchuva plant or peach tree, counting the number of females and males on each type of fruit treatment and annotating the type of behavior exhibited by the flies on the plant or on cage walls/roof following a scan-sampling protocol [34]. Behaviors were classified as resting, cleaning, fruit-foraging, ovipositing, feeding or stuck on the fruit surface. Each observation period lasted 15 min, during which the entire plant was observed, as the observer walked slowly around it. The remaining 45 min were devoted to resting and to inspecting the cage walls or Uchuva plants (peach trees) for the presence of predators (in case one was found it was captured and removed from the cage). This procedure was repeated eight times per day during the three-day observation period.

### 2.3. Forced Infestation under Artificial Laboratory Conditions

**Figure 4:**
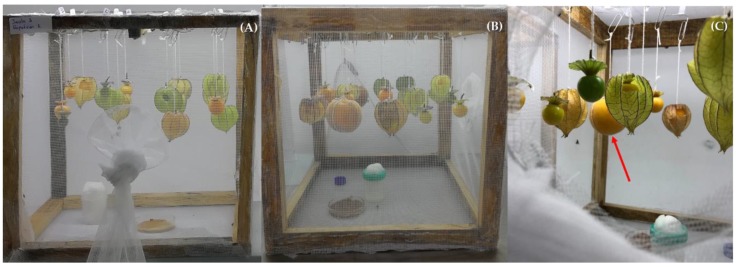
Laboratory cages with treatments used in two types of choice experiments. (**A**) Cage with various types of *P. peruviana* fruit hung from the roof; (**B**) cage with both *P. peruviana* and *P. persica* fruit hung from the roof (also representing various types of fruit and degrees of ripeness); (**C**) view inside cage to better visualize fruit conditions tested. Note the peach (red arrow) next to various Uchuvas devoid of the calyx or with a “window” cut open to allow female flies to access the fruit.

Given that not a single Uchuva fruit was ever found to be infested under completely natural field conditions and also under semi-natural field-cage conditions in the June 2018 tests (between January–March 2019 we learned that this also applied to the December 2018 tests), two additional laboratory experiments were designed to determine, if under highly artificial conditions, *C*. *capitata* females of wild origin would be able to oviposit into Uchuvas and if so, whether the eggs would hatch, develop larvae and pupate and adults emerge. *Prunus persica* cv ‘Jarillo’ fruits were used as positive controls. The studies were run in December 2018 in a specially conditioned laboratory in the headquarters of the local ICA office in the City of Pamplona, Norte de Santander, Colombia. Two sets of experiments were run: 1) A choice experiment combining Uchuvas and peaches in the same cage, and 2) a choice experiment with only Uchuvas of varying degrees of ripeness, calyx condition and in one treatment, damaged fruit (details in Figure 4). We also prepared additional cages into which we only introduced peaches of the same three degrees of ripeness used in the choice experiment that combined Uchuvas and peaches. The latter to experimentally confirm the preference of Medfly females for a certain degree of ripeness in peaches, and to ensure that our test insects were able to lay viable eggs.

In the Uchuva-only experiment, we tested the same 15 treatments as in field cages (Figure 3A), plus an additional cage designed to place the Uchuva fruit under the most extreme risk of oviposition activity by *C*. *capitata* females. Treatment 16 (Figure 3A) consisted of a fully ripe Uchuva (ripeness level six of Figure 3A) which was injured in the middle (using a surgical scalpel, we performed a fine cut into the skin that slightly penetrated the pulp (fruit indicated by red arrow in the lower right of Figure 3A)). The injury was done to allow females to better secure themselves on the fruit and potentially insert their aculeus for oviposition, as otherwise the surface resins/waxes caused females, or their extruded aculei, to slip off the fruit surface. In addition to these 16 types of Uchuvas (treatments), in the case of the second-choice experiment, three peaches (each one representing a distinctive degree of ripeness) were added to the 16 Uchuvas, totaling 19 fruit per cage (Figure 4). In the choice experiment considering only peaches, three fruit of differing ripeness were hung in the respective cages (green-ripe, ripe and very ripe)

The bioassay consisted of releasing 32 (two female flies per fruit) sexually–mature, gravid females and 16 sexually mature males of 11–25 days of age, in the Uchuva-only choice experiment and 38 females and 19 males in the Uchuva and peach choice experiment (also two females per fruit), into 30 × 30 × 30 cm cages held together with wood frames and covered with white Tulle cloth (Figure 4A,B). In the choice experiment considering only peaches, six females (two per fruit) and three males were released. In each cage, we carefully hung from the roof one each of the types of *P*. *peruviana* or peach fruit we wanted to expose to the oviposition activity of *C*. *capitata* (Figure 4C). The posterior part of the cages was covered with Plexiglas to facilitate observations. Tests were replicated 12 times, and on each occasion, the distribution of fruit hanging from the roof was modified by using a random number generator (each fruit had a numbered tag identifying the treatment). In every replicated experimental unit, fruits were exposed continuously to the oviposition activity of females for 72 h. Considering the 12 replicates per choice experiment (only Uchuvas, Uchuvas with peaches and peaches alone), a total of 384 Uchuvas and 72 peaches were exposed to the oviposition activity of a total of 912 sexually mature, gravid *C*. *capitata* females. We were unable to make systematic observations on fly behavior from outside of the cages, as too many activities occurred simultaneously, but a few scan-sampling events were sufficient to identify differences in fly behavior.

Once the exposure period was completed, Uchuvas and peaches were placed individually (one fruit per container) in 350 mL and 470–1000 mL plastic containers, respectively, containing vermiculite as pupation medium. In the case of Uchuvas, containers were inspected at 15 day intervals over a three-month period. In the case of peaches, as they rotted readily, containers were inspected for the first time at day seven, and thereafter every three days until all larvae had pupated or fruit had rotted completely.

We note that during October 2017 one of us (MA), with the help of Juan Camilo Rodríguez Guaqueta (Instituto Colombiano Agropecuario, ICA), and under the auspices of Boris Orduz (also ICA), was able to make preliminary observations on the behavior of laboratory-reared, mated/gravid *C*. *capitata* females around very ripe Uchuva fruit that were totally devoid of the husk. These fruits were placed on the floor of a Plexiglas cage. These preliminary observations carried out in Bogotá, Colombia, yielded helpful insights into the role of surface resins/waxes in the inhibition of landings on fruit. On the rare occasions that surface waxes did not repel flies, oviposition attempts by *C*. *capitata* females in Uchuvas were observed. This experience helped us better design the formal laboratory choice tests performed during December 2018, by exposing wild females to a wide array of options.

### 2.4. Volatile Collections in P. peruviana Branches

Considering the fact that during the November 2018 field-enclosure preliminary tests, we observed what appeared to be signs of intoxication in some *C. capitata* adult individuals when sunlight impinged directly on Uchuva plants. During this behavior flies started to rub their legs and clean their wings and on occasions ended ventral side up on leaf surfaces (Figure S3). We presumed that Uchuva leaf or branch volatiles could be causing this. Consequently, we collected volatiles directly in the field from branches of *P. peruviana* plants of the same size and shape, located close to our field enclosures. We tested three conditions: a) intact branches, b) newly pruned branches and c) branches pruned and allowed to “heal wounds” for 24 h. As was the case in the plants used for our field-enclosure exposures, pruning consisted of cutting some flowers, floral buds, fruit, fruit calyxes and branch parts.

To collect volatiles, three branches of each plant condition were completely covered with adapted turkey oven bags (Reynolds^®^, Lake Forest, IL, USA) to apply a dynamic extraction method. A purified airflow (1 L/min) was driven through the collection chambers and odors were collected in a HayeSep Q (VCT-1/4-3-HSQ-P, 0.02 g; 60/80 mesh; ARS) filter placed in the collection port (vacuum system) of the bags for three hours. Volatile compounds were eluted from the adsorbent with 0.4 mL of dichloromethane (≥ 99.9% GC grade, Sigma Aldrich, Lyon, FR) and stored at −80 °C prior to analysis.

Plant volatile compounds were analyzed using a gas chromatograph (GC-2010 Plus, Shimadzu, Canby, OR, USA) coupled to a mass spectrometer (QP-2010 Ultrasystem, Shimadzu) with a J & W HP 5MS, Agilent column (30 m, 0.25 mm internal diameter, 0.25 μm film thickness). The GC-MS was programmed with three temperature ramps. The oven initial temperature was at 50 °C, which increased at 10 °C/min intervals up to 180 °C, then at 1.5 °C/min intervals until reaching 200 °C, and was then held for 2 min at this temperature, then from 200 °C the temperature was increased 6 °C/min until reaching 290 °C and remained at this temperature for 1 min. The temperature for the interphase was 290 °C. In addition, 1 mL of the sample was also injected at 280 °C in the splitless mode.

Identification of compounds was confirmed by reference standards when they were commercially available; otherwise tentative identification was obtained via a mass spectrum comparison of compounds with reference standards registered in the National Institute of Standards and Technology (NIST) library.

### 2.5. Data Analyses

The mean percentage of events related to each behavior recorded inside Uchuva and peach field cages were analyzed by non-parametric Mann–Whitney test, considering each observation day per cage as a replicate. To determine female preferences for peaches of varying degrees of ripeness (in the case of Uchuvas not a single oviposition event was recorded) a correlation analysis was performed. To compare the proportion of infested fruit among the different ripening stages in field and laboratory studies, a non-parametric Kruskal–Wallis ANOVA was performed. Finally, we compared the weight of pupae that originated from field vs lab infested fruit by t-test for independent samples. All analyses were performed using Statistica^©^ software, Version 10 (StatSoft Inc., Tulsa, OK, USA) [35].

With respect to Probit 9 calculations, we based our computations on those described by Follet and Hennesey [36]. Based on these authors, the number of fruits sampled is used to determine the confidence level by using the following formula:

*C* = 1 − (1 − *p_u_*)*^n^*

where *p_u_* represents the acceptable level of survivorship and *n* is the number of fruit tested.

## 3. Results

Considering our three-year field sampling effort and the field-cage and laboratory studies, we were unable to document a single case of the successful development of *C*. *capitata* in *P*. *peruviana*. In contrast, we obtained many Medflies from peaches collected in the field (from wild hosts, orchards and field-enclosed trees) or those that had been artificially infested in the laboratory.

### 3.1. Fruit Sampling in the Field and Adult Fly Trapping

None of the 92,503 individual *P*. *peruviana* fruit (630 kg) collected directly in the field in 34 commercial Uchuva plantations (from fruit-bearing plants and from the ground) in the Departamento Norte de Santander, Colombia between 2016 and 2018, yielded a single *C*. *capitata* pupa. Similarly, none of the 5629 (32.45 kg) individual *P*. *peruviana* fruit collected from ‘feral’ Uchuva plants growing wild in the same region, or from the ground, yielded any Medfly pupae. This contrasts sharply with the 17,611 pupae collected from 452.3 kg of peaches in the same region and used as sources for *C*. *capitata* adults for our field-cage and laboratory studies. That is, even though *C*. *capitata* was present in high numbers in the region, females did not infest Uchuvas. The fact that Medflies were highly abundant in the study region was also documented by the large numbers of adults captured in the 477 Jackson traps placed along our trapping routes in the study region (Figure S2B). The mean number of flies/trap/day over the entire 2015–2017 trapping period was 1.28 ± 0.04 SE, with a peak of 4.37 on week 52 of year 2017.

The prevalence of fruit infestation by *C*. *capitata* in a variety of hosts in the region, including peaches, was also documented in our large scale, weekly fruit sampling scheme over a three-year period. Of the 54 fruit species sampled, nine (peach, coffee, guava, pepper, feijoa, fig, apple, loquat and orange) were infested by *C*. *capitata* (Table S1).

#### Probit 9 Calculations

We followed the following formula as recommended by Follet and Hennessey [36] to calculate Confidence Level: *C* = 1 − (1 − *p_u_*)*^n^*. Having sampled a total of 98,312 fruit (*n*) in the field without even one being infested (*p_u_* or acceptable survival level - 100 − 99.9968/100), we reached the Probit 9 level with 99.9968% efficacy and a confidence level of 96%.

### 3.2. Forced Infestation Studies under Field-Cage Conditions

**Figure 5:**
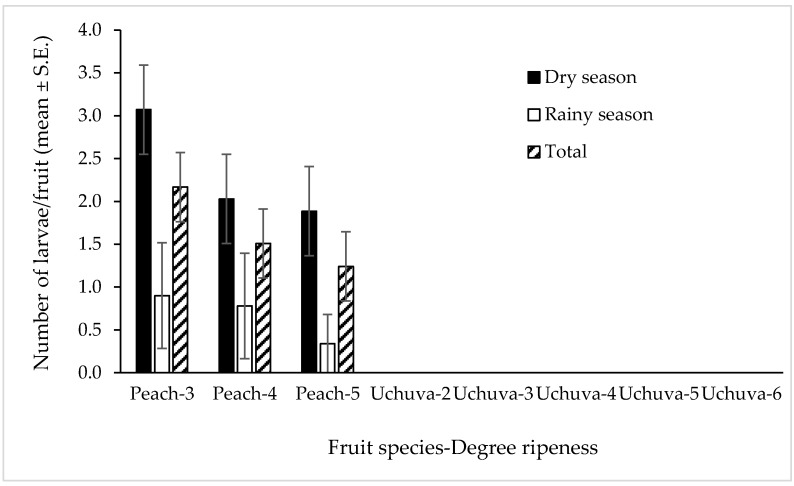
Infestation levels (mean ± S.E. number of larvae per fruit) in peaches infested by *C. capitata* according to the degree of fruit ripeness in infestation assays in field cages during the rainy and dry seasons.

We were unable to detect a single infested fruit in any of the 600 Uchuvas exposed to the oviposition activity of 1800 *C*. *capitata* females in June 2018 (rainy season) or December 2018 (dry season) experiments. In contrast, of the 360 peaches, of three different degrees of ripeness, exposed under the same experimental and ambient conditions to 1080 gravid *C*. *capitata* females in the mirror tests run as controls to guarantee that the flies released into the field cages were active and able to lay fertile eggs, 75 fruit (20.83%) were infested and yielded 590 pupae. Of these, 101 pupae of *C*. *capitata* originated from the two peach trees exposed in June and 489 from the single tree exposed in late November 2018 and the two trees exposed in December 2018. We were able to detect a statistically significant difference in the degree of infestation (i.e., number of larvae per fruit) among the different degrees of ripening in peaches in either season (Kruskal–Wallis ANOVA: H = 1.83, df = 2, 150; *p* = 0.401 for June and H = 2.18, df = 2, 210; *p* = 0.336 for November/December), although the least ripe peaches tended to have higher levels of infestation in both seasons (Figure 5). In June (rainy season) of 21 peaches that were infested, 10 (47.6%), 6 (28.6%) and 5 (23.8%) corresponded to ripeness levels three, four and five, respectively. In the case of November/December, of the 54 peaches that were infested, 22 (40.74%), 17 (31.48%) and 15 (27.78%) were of ripeness levels three, four and five, respectively.

#### Fly Behavioral Patterns in Field-Cages

In late November 2018, we noticed that when temperatures started to rise and sunlight impinged sideways into the enclosed *P*. *peruviana* plants, some flies began to exhibit signs of intoxication. That is, they cleaned their bodies or remained quiescent and, in some cases, started to walk very slowly on the surface of leaves eventually turning ventral side up (Figure S3). Surprisingly to us, when these flies were placed in a clean cage and returned in the afternoon to the laboratory, they all recovered and became active again and lived many days without any apparent damage. We postulated that these presumed signs of intoxication could be the result of having pruned/removed flower buds, flowers and some branches/leaves in the water-stressed Uchuvas. Therefore, to facilitate observations the same day that we released flies into the cage, we decided to collect volatiles in contiguous *P*. *peruviana* plants treated identically in search of possible repellents or toxicants. Importantly, and based on the possible intoxication, we ran the formal dry season experiment in early December 2018 allowing plants to rest and heal pruning “wounds” for 72 h prior to releasing test flies, as had been done in June 2018. Under these conditions, flies did not exhibit any signs of intoxication, as had been the case in the rainy season when we worked with Uchuva plants that did not suffer water stress.

**Figure 6:**
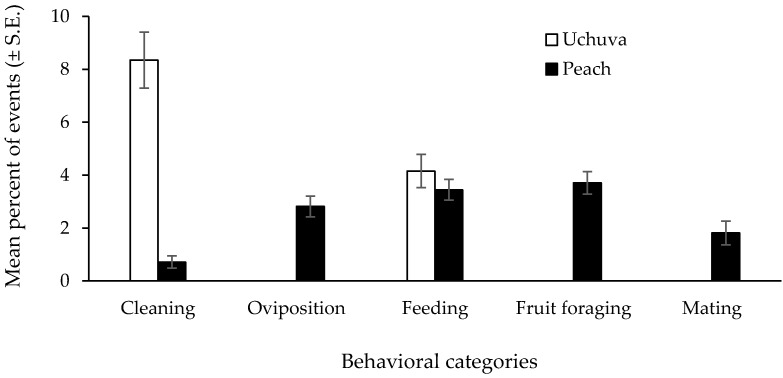
Behavioral events recorded in *P. peruviana* and *P. persica* plants/trees in field-cage studies. Since most of the recorded events inside field cages were represented by acquiescent behavior (90.2% in peach trees and 98.1% in Uchuva plants; please see discussion of this phenomenon in the text), the graph only depicts the distribution of the behavioral events related to the remaining behaviors recorded in each type of cage.

The activity patterns of Medflies observed in the December 2018 dry season observation period are summarized in Figure 6. A total of 20,103 behavioral events were recorded, 15,663 (77.9%) in the four Uchuva plants and 4440 (22.1%) in the three peach trees as we included the tree used in late November. In both cases, resting behavior (possibly also cleaning that was not easily detected from a distance) was the most frequently recorded: 90.2% (n = 4004) in peaches and 98.1% (n = 15,358) in Uchuvas. The remaining proportion of behavioral events was distributed as shown in Figure 6, with no oviposition behavior ever observed in Uchuva plants. Overall, Medfly females exhibited statistically different behavioral patterns in peach trees when compared to Uchuva plants (Figure 6). Importantly, in the case of Uchuvas, cleaning behavior was significantly different (Mann–Whitney test: Z = −3.80, *p* = 0.0001) when compared to what was observed in the enclosed peach trees (Figure 6). As mentioned before, flies appeared irritated by the volatiles emitted by both Uchuva plants used in the two-preliminary end-of-November training sessions, exhibiting on occasions, signs of temporary intoxication In the case of feeding behavior, the proportion of events observed was not statistically higher in peaches when compared to Uchuvas (Mann–Whitney test: Z= −0.11, *p* = 0.915). Finally, with respect to oviposition, fruit foraging and mating, differences between peaches and Uchuvas were highly significant (Mann–Whitney test: Z = 3.80, *p* = 0.0001 in all cases). In the case of female oviposition activity in peaches of varying degrees of ripeness, no statistically different preference among fruit of the three degrees of ripeness was detected (r = −0.008, *p* = 0.91).

### 3.3. Laboratory Studies

As was the case in the field-cage studies, none of the 384 Uchuvas of varying degrees of ripeness and damage to the calyx and skin of the fruit, became infested following exposure to the oviposition activity of gravid *C*. *capitata* females. This contrasts with the 205 pupae obtained from the 36 peaches exposed together with Uchuvas in the choice experiment. The infestation pattern of naturally attached peaches exposed to *C*. *capitata* females in field enclosures and of harvested peaches exposed in small, laboratory cages was very similar. In addition, the mean weight of pupae originating from field-collected peaches that were naturally infested by *C*. *capitata* females (9.63 ± 0.39 mg, SE, N = 20) and pupae originating from peaches that were infested by the females (also of wild origin) in the laboratory choice tests (10.79 ± 0.83 mg, SE, N = 85, maturity stage three) were statistically similar (t-test = −0.67; df = 103; *p* = 0.51). That is, the flies used in the experiments were of similar size and weight as flies reared from naturally infested peaches.

We note that in these highly artificial experiments, and as observed in preliminary studies in 2017, flies seemed to be repelled by unidentified volatiles emitted by the surface resins/waxes covering the Uchuva fruit or flowing from the fruit itself, as few individuals landed on Uchuvas, and when this occurred, they quickly left. Females were not stimulated into host finding or oviposition behavior on Uchuvas, which contrasted with the peaches on which they readily landed, apparently recognizing the distinctive odors emitted by the three types of fruit available to them. In the case of peaches, we found no significant differences with respect to levels of infestation (Kruskal–Wallis ANOVA: H = 1.69, df = 2, 36; *p* = 0.428 for the choice experiment with peaches and Uchuvas mixed together, and H = 2.12, df = 2, 36; *p* = 0.347 for the choice experiment with peaches kept separately). In the few scan observations we made on the 24 cages where the choice experiments involving Uchuvas were being conducted, not a single oviposition event was observed, even in the injured fruit. We note, however, that in the preliminary observations during 2017 with lab-reared flies in the Bogotá ICA quarantine facility (MA and JCRG, unpublished observations), in the few cases where landings on fruit were observed and oviposition attempts noticed, females could not secure themselves on fruit as they slipped due to the surface resins/waxes or when they managed to support themselves, they were unable to insert the aculeus as it also often slipped due to the surface resins/waxes.

### 3.4. Volatile Collections

We were able to identify 32 volatile compounds in the volatile collections made directly in the field (Table 2). Not surprisingly, the freshly pruned/damaged branches emitted the greatest number of volatiles (23) compared with the intact ones (20), and the branches that had been pruned/damaged 24 h prior to volatile collections (17), which mimicked the conditions in the two cages used in late November to train observers. Also, freshly pruned branches and branches pruned 24 h prior to volatile collections, emitted volatiles in higher concentrations when compared to intact plants.

## 4. Discussion

Clearly, *P*. *peruviana* exhibited a total degree of resistance to the attack of *C*. *capitata* under natural field and experimental conditions in Colombia (or there was behavioral non-preference by the insect to the plant and fruit). To begin, in 98,132 fruit collected in the field (from standing plants (97.8%) and from the ground (2.2%)) in commercial plantations (94.27%) and from ‘feral’ fruit growing the wild (5.73%), we were unable to detect a single infested fruit. With this amount of fruit sampled, we reached the Probit 9 level with 99.9968% efficacy and a confidence level of 96%, which for quarantine purposes is highly relevant [36]. We highlight the fact that all Uchuvas were collected in a region of Colombia where *C*. *capitata* populations are very high and many natural host plants are present, all of which were heavily infested (Figure S2A,B). This represents part of the solid evidence we present here indicating that *P*. *peruviana* cannot be considered a natural host based on the strict criteria described by Aluja and Mangan [4] and the ISPM-37-3 FAO guidelines [5]. In addition, *P*. *peruviana* fruit that were exposed to the oviposition activities of gravid *C*. *capitata* females in naturally-growing, caged, fruit-bearing Uchuva plants, or under highly artificial conditions in the laboratory were also not infested, even when they were even intentionally damaged (horizontal slit cut with a scalp (Figure 3A)) to facilitate female oviposition. On top of this, we observed signs of repellence/toxicity to ovipositing females from odors possibly emitted by the surface resins or waxes present on the Uchuva fruit, and signs of repellence or intoxication to adult *C*. *capitata* individuals via volatiles emitted by the plant. In what follows, we discuss these findings and consider what is known concerning the toxicity of *P*. *peruviana* to herbivores.

We were able to identify three layers of resistance in *P*. *peruviana* to the attack of ovipositing *C*. *capitata* females: (1) the calyx or husk; (2) if holes in the husk were created by lepidopteran larvae in the field, either chemicals in the pulp or surface resins/waxes in ripe fruit hindered larval development or aculeus insertion by *C*. *capitata* females; (3) repellent or toxic volatiles emitted by the fruit and the foliage. Of these, the most important hurdles a female fly faces are the husk and the surface resins/waxes, and in the case of larvae, the toxic chemicals in the fruit. Alternatively, or in addition to resistance, the phenomenon we observed could also be interpreted as a case of “behavioral non-preference” given that we could not directly test the resistance of the fruit to feeding larvae as not a single fruit collected in the field or used in our experiments turned out to be infested. However, as we will discuss in what follows, there are published studies documenting that larvae are indeed intoxicated by the chemicals in *P*. *peruviana* fruit.

Given that the aculeus of *C*. *capitata* has a mean length of 1.33 ± 0.019 mm [37], even if a female was able to pierce the calyx, it would not be able to reach the fruit inside to lay an egg (mean distances from the fruit border to the bottom and middle parts of the calyx wall are 16.1 ± 0.5 SE and 9.4 ± 0.5 SE mm, respectively (Figure 1A). In cases where gravid females flew or walked through the calyx in fruit on which this structure was artificially damaged, so as to mimic the feeding activity of a moth larva in the field (Figure 1), or in fruit in which the calyx had been totally removed, we noticed that, upon landing, females were apparently repelled by volatiles emitted by the fruit or by fruit surface resins/waxes, supporting the behavioral non-preference concept. On the rare occasions where landing occurred, females were unable to secure themselves on the fruit surface to insert their aculeus, as they often slipped. Even when they were able to find some support on the internal calyx surface close to the peduncle, many times their aculeus also slipped, thus preventing successful insertion into the fruit. For this reason, we artificially damaged fruit in one of our treatments by performing a lateral incision using a surgical scalpel (the fruit indicated by a red arrow in Figure 3A). Not even under these highly artificial conditions in the laboratory were we able to document infestations. In comparison, although present in the same cages, next to Uchuvas of varying degrees of ripeness and with damaged husks (Figure 4B,C), peaches invariably yielded abundant pupae and adults.

The third level of resistance (and/or behavioral non-preference), detected in *P*. *peruviana* is the repellency or toxicity of both aerial parts and fruit to *C*. *capitata* and other insects. This is an important issue when considering its status as a host. Veleiro et al. [31] reviewed the chemistry of over 300 withanolides that are widespread within the Solanaceae, highlighting the fact that some are known as feeding deterrents in insects. Aerial parts of *P*. *peruviana* contain several withanolides including withanolide E and 4-β-hydroxywithanolide E, that are either directly toxic to *C*. *capitata* larvae or delay larval development and duration of the pupal stage [27]. The toxicity of withanolides (triterpenoids) from aerial parts of *Salpichroa origanifolia* (Lam.) Baill. (Solanaceae as is the case with *P*. *peruviana*) to *C*. *capitata* had been reported previously by Bado et al. [26]. These authors exposed Medfly larvae to salpichrolide A–G obtained from leaves of *S*. *origanifolia* and report that salpichrolide B caused high (95%) larval mortality. Adults exposed to the withanolides via the water they consumed, also experienced a high prevalence of mortality [26]. It is true that *C*. *capitata* larvae do not feed on leaves but rather develop inside fruit, by consuming the pulp. In this respect, as early as 1993, Bauman and Meier [28] reported on the chemical defense properties of withanolides during fruit development in *P*. *peruviana*. They identified the same withanolides in both the calyx and the berry with which Cirigliano et al. [27] worked. That is, the fruits of Uchuva were also found to contain these types of toxic chemicals to insects, a fact later confirmed by many authors. For example, Llano et al. [38], working on conventional and organic Uchuvas, identified Physagulin D, a whitanolide. In addition to withanolides, *P*. *peruviana* fruits contain polyphenols, physalins and phytosterols which are known as cholesterol-reducing, antioxidant, anti-diabetic, anti-inflammatory, antimicrobial, antitumoral, hepatoprotective, immunomodulatory, analgesic, antiparasitic and diuretic compounds in humans [8,39,40,41,42].

In addition to these layers of resistance or behavioral non-preference, we observed that when *C*. *capitata* adults were released into cages covering an Uchuva plant in the field during the dry season (experiencing hydric stress), some individuals exhibited signs of irritation or possible intoxication (Figure S3). Consistent with this, some of the chemicals identified in the volatiles collected directly from Uchuva plants, such as 1-Nonene, produce an irritating vapor (PubChem, https://cameochemicals.noaa.gov/chemical/8913). Others such as beta-caryophyllene, α-pinene, phellandrene, humulene, ylangene and ethyl octanoate which were detected in higher proportions in the volatiles from freshly damaged plants, or those pruned 24 h prior to volatile collections (Table 2), have been reported by other authors as repellent or toxic to various other insects [43,44,45,46,47,48,49]. That is, some of the volatiles we identified from undamaged or damaged *P*. *peruviana* branches are indeed known to repel or kill insects. We note however that the observation that flies spent most of their time resting and cleaning in field-cage studies is not uncommon. For example, Aluja et al. [34] working with *Anastrepha striata* Schiner in a large field cage enclosing potted guava, sapodilla and citrus trees, reported that in 564 h of observations, 32,886 resting events were recorded. Years later, Aluja et al. [50] wrote, after studying the basic patterns of behavior of *Rhagoletis turpiniae* Hernández, “in our study, the majority of *R*. *turpiniae* adults spent most of the observation period resting on the host plant”.

With respect to the repellent or toxic volatiles emanating from Uchuva plants, it is likely that this phenomenon was in part triggered by the hydric stress the plants experienced during the dry-season observation period. As noted in the Materials and Methods section, the field site had no artificial watering system, and no rainfall was recorded during the dry season study period. Stressed plants emit more defensive volatiles than unstressed ones [51], and this could have been the case inside the experimental cages, as most signs of what appeared to be intoxication were observed when the incident sunlight was at a low angle to foliage, which may have raised the temperature and the stress condition of the Uchuva plants. The same phenomenon was observed in the enclosed branches from which volatiles were collected from those branches were the damage (i.e., cutting excess branches or leaves) was inflicted recently (immediately and at 24 h after injury). But importantly, in the June and December 2018 studies, in which we waited three days after pruning the plants before releasing flies into the cage, no flies showed signs of intoxication. In conclusion, the volatile-toxicity phenomenon is most likely related to hydric stress conditions and sheds light on the potential toxicity/repellency of Uchuva volatiles to *C*. *capitata* (and likely other insects) during certain environmental conditions. In future studies, we will try to better understand the mechanism by which the Medfly is repelled by the plant, as there are many potential practical applications related to our finding.

Finally, we consider the fact that we reached the Probit 9 level with 99.9968% efficacy and a confidence level of 96% based on 98,132 fruits collected in the field that were not infested, which for quarantine purposes is highly relevant, particularly in the context of host plant determination [36]. Non-host status determination could be theoretically reached based on this criterion alone. But here, besides the Probit 9 computations, we have amassed large amounts of data using a multilayered approach (i.e., biological, experimental, probabilistic and literature records) that yielded consistent evidence, all pointing in the same direction. In addition to this multi-perspective evidence, according to official figures from the Sistema de Información Sanitaria para Importación y Exportación de Productos Agrícolas y Pecuarios (SISPAP)—Instituto Colombiano Agropecuario (ICA) [52], Colombia has exported in the past four years 1,368,423 kg of Uchuva to the USA. In addition, another 22,279,047 kg of Uchuva from Colombia have been exported to another 35 countries throughout the world between 2015 and 2018 (Supplementary Table S2). Considering the total volume exported to all these countries including the USA (23,647 tons), not a single fruit has so far been reported as infested by *C*. *capitata*. This is noteworthy as 23,647 tons are equivalent to ca. 3500 million individual fruit assuming a mean weight of 6.8 g per fruit. This represents massive non-experimental data on which a non-host decision could also be partially based.

## 5. Conclusions

We were not able to find a single infested *P*. *peruviana* fruit under completely natural field conditions, in commercial Uchuva plantations, in field-cage experiments and in highly artificial laboratory studies. In addition, we identified possible volatile repellents or toxicants emanating from damaged Uchuva branches, which adds to literature reports indicating that chemicals in *P*. *peruviana* fruit are toxic to *C*. *capitata* larvae [26,27]. Taken together, the data lead us to conclude that *P*. *peruviana* is not a natural host of *C*. *capitata* and, at least under our experimental conditions, cannot even be considered a conditional host based on the criteria described by Aluja and Mangan [4] and the ISPM-37-3 FAO guidelines [5]. As noted in the introduction, the previous reports indicating that *C*. *capitata* can infest *P*. *peruviana* in Hawaii may be debatable. Also, based on the information provided in one of the publications by Liquido et al. [23], we were able to infer that the fruit in Hawaii are extremely small compared to the ones we collected in nature and commercial plantations in Colombia (2.4 vs 6.8 g (Table 1)). Perhaps Nakagawa et al. [21] and Liquido et al. [22] worked with locally adapted *P*. *peruviana*/*C*. *capitata* cultivars/races, or with weak, underdeveloped fruit or fruit that had been severely damaged by birds or insects, which may have resulted in a loss of the natural toxicity that Uchuvas exhibit under other circumstances. In addition, one of the same authors [23] and another independent group [24], also working in Hawaii more recently, were unable to confirm those original reports. Therefore, based on the mass of evidence accrued here, it can be concluded that *P*. *peruviana* is not a host for *C*. *capitata*.

## Figures and Tables

**Table 1:** *Physalis peruviana* (Uchuva) sampled in commercial plantations and nature over a three-year period in the Departamento Norte de Santander, Colombia (details in Figure S2).

Year	Fruit	Sampling Part	Kg of Fruit	Total no. Fruit
Total 2016			184.6	33,079
	Commercial Uchuva plantations		171.4	30,537
		Plant	166.2	29,052
		Soil	5.2	1485
	“Feral” Uchuva plants	Plant	13.2	2542
Total 2017			445.4	60,221
	Commercial Uchuva plantations		427.0	57,317
		Plant	425.0	57,085
		Soil	1.9	232
	“Feral” Uchuva plants		18.4	2904
		Plant	15.4	2408
		Soil	3.0	496
Total 2018			32.6	4832
	Commercial Uchuva plantations	Plant	31.7	4649
	“Feral” Uchuva plants	Plant	0.9	183
Total			662.5	98,132

**Table 2:** Chemical compounds identified in intact and damaged *P. peruviana* fruit-bearing branches.

Compounds	Intact Branches		Recently Pruned Branches		Branches Pruned 24 h Prior to Volatile Collection	
	RT	Relative Area	RT	Relative Area	RT	Relative Area
Methyl isopentanoate			3.310	16,473,762		
?	3.622	160,694,822	3.616	95,356,938		
?	3.81	26,810,425				
Methyl 2,3-dimethylbutanoate			4.310	13,745,891.3		
3-Hexen-1-ol, (Z) *			4.394	610,92,890.7		
trans-2-Hexenol			4.534	26,142,420.7		
Octylcyclopropane/1-Nonene					4.879	8,460,853
Cyclene	5.472	7,052,744	5.467	44,547,522	5.462	61,727,006.7
alpha-Pinene *	5.650	7,101,577	5.645	14,490,525	5.639	28,033,637
?	5.905	32,962,783.5	5.900	38,290,510	5.896	12,457,773
beta-Phellandrene			6.273	14,704,868.5	6.269	19,964,512.5
Butyl butanoate	6.537	193,621,106	6.532	177,265,806		
sec-Amyl butyrate	7.549	26,157,546	7.543	34,564,042		
?	8.134	36,909,580.5				
Methyl octanoate			8.590	8,737,293.5		
Ethyl octanoate *	9.726	91,124,017.5	9.720	47,461,136	9.717	34,196,081
delta-EIemene					12.028	9,593,930.5
Ylangene	12.579	42,290,590	12.576	52,852,747.3	12.573	64,819,090.7
alfa-Copaene *			12.645	22,192,225.7	12.640	30,216,856.3
?	12.657	18,306,226				
beta-Bourbonene	12.810	116,855,780	12.815	113,935,875	12.808	142,049,337
beta-Cubebene	13.285	37,045,601.3				
Caryophyllene *	13.323	37,115,958.7	13.321	145,049,228	13.318	193,915,800
beta-copaene *	13.433	28,194,287.3	13.430	31,627,144	13.427	28,854,676.3
gamma-Cadinene/beta Cubebene	13.489	49,388,138.3	13.488	68,758,807	13.487	79,022,234.3
gamma-Muurolene/Eremophilene	13.660	18,646,364.5	13.656	14,572,749.3	13.652	17,154,062
Humulene *	13.812	17,520,372	13.815	21,593,178.7	13.813	25,674,739
Germacrene D	14.227	252,723,612	14.224	251,971,967	14.220	253,423,376
gamma-Cadinene	14.7	17,871,571	14.697	19,851,101.7	14.693	14,956,966.7

* Compounds verified with pure standards. RT = Retention time. Relative areas calculated according to the total area of the listed compounds. ? Indicates unknown compounds or ones we were unable to identify.

## Supplementary Materials

The following are available online at https://www.mdpi.com/2075-4450/10/12/434/s1, Figure S1: Study region indicating location within Colombia and then within the Departamento Norte de Santander where the study was conducted (Cácota and Chitagá), Figure S2: (A) Fruit collection and (B) trapping routes in the Departamento Norte de Santander, Colombia. The study site was close to the border of Venezuela, ca. 90 min away from the city of Cúcuta, Figure S3: *Ceratitis capitata* female exhibiting signs of intoxication on top of an Uchuva leaf, Table S1: Plant species sampled over a three-year period in the Departamento Norte de Santander (details on sampling routes are provided in Figure S2) to identify *Ceratitis capitata* local hosts and in the case of positive findings, degree of infestation (number of larvae/kg fruit), Table S2: *Physalis peruviana* export statistics from Colombia to 36 countries, including the US from 2015 to 2018 (Source: Sistema de Información Sanitaria para Importación y Exportación de Productos Agrícolas y Pecuarios-Instituto Colombiano Agropecuario [SISPAP-2019]).
