# Supplementary material for: Physalis peruviana L. (Solanaceae) Is Not a Host of Ceratitis capitata (Diptera: Tephritidae): Evidence from Multi-Year Field and Laboratory Studies in Colombia
Source: Insects. 2019 Dec 4;10(12):434. doi: 10.3390/insects10120434 (PMC6956068; doi:10.3390/insects10120434)
Supplement: Supplementary file 1 [file insects-10-00434-s001.zip › Supplemental Figure 2.pptx]

## Slide 1
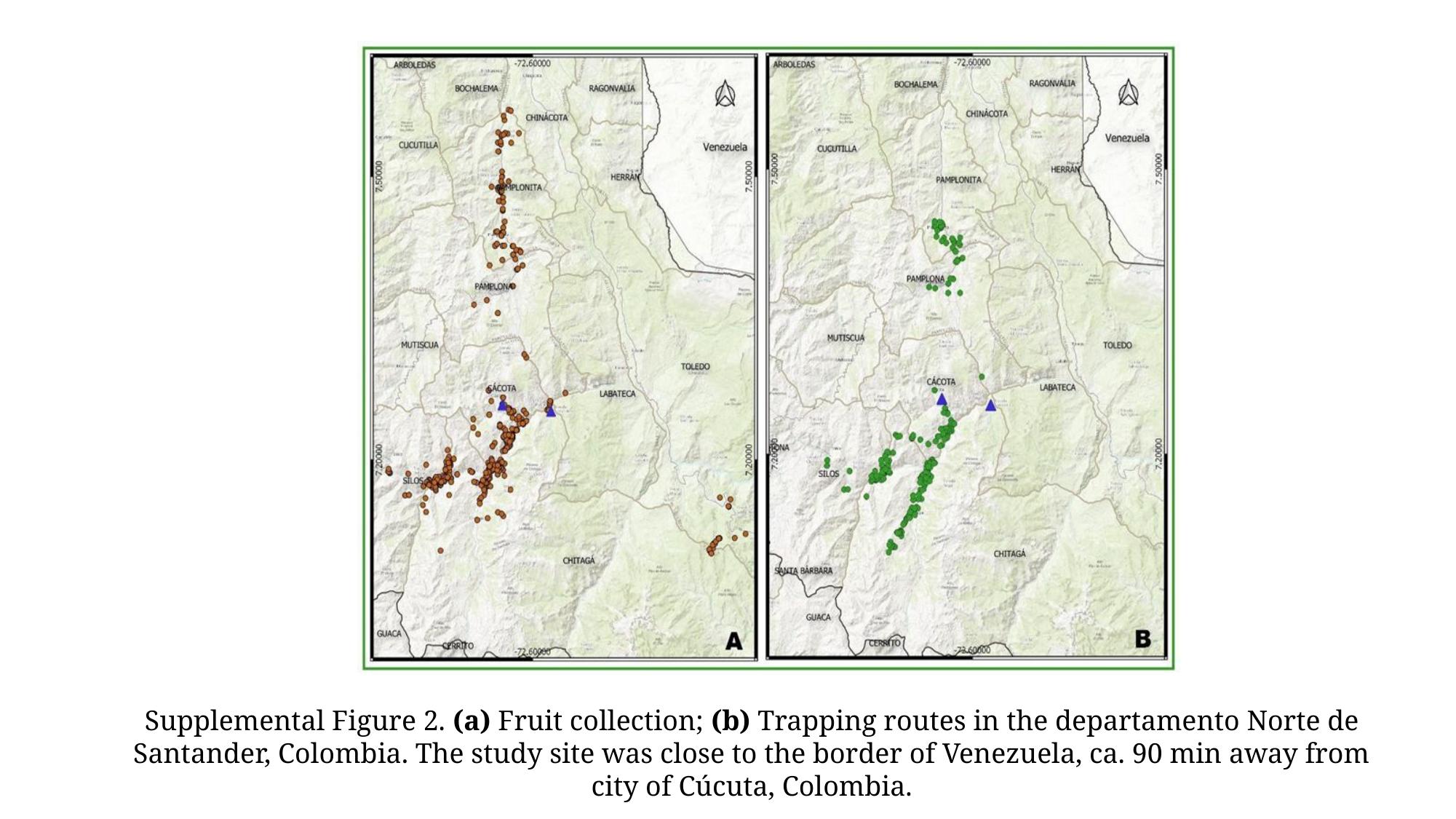

Supplemental Figure 2. (a) Fruit collection; (b) Trapping routes in the departamento Norte de Santander, Colombia. The study site was close to the border of Venezuela, ca. 90 min away from city of Cúcuta, Colombia.
