# Supplementary material for: Physalis peruviana L. (Solanaceae) Is Not a Host of Ceratitis capitata (Diptera: Tephritidae): Evidence from Multi-Year Field and Laboratory Studies in Colombia
Source: Insects. 2019 Dec 4;10(12):434. doi: 10.3390/insects10120434 (PMC6956068; doi:10.3390/insects10120434)
Supplement: Supplementary file 1 [file insects-10-00434-s001.zip › Supplemental Figure 3.pptx]

## Slide 1
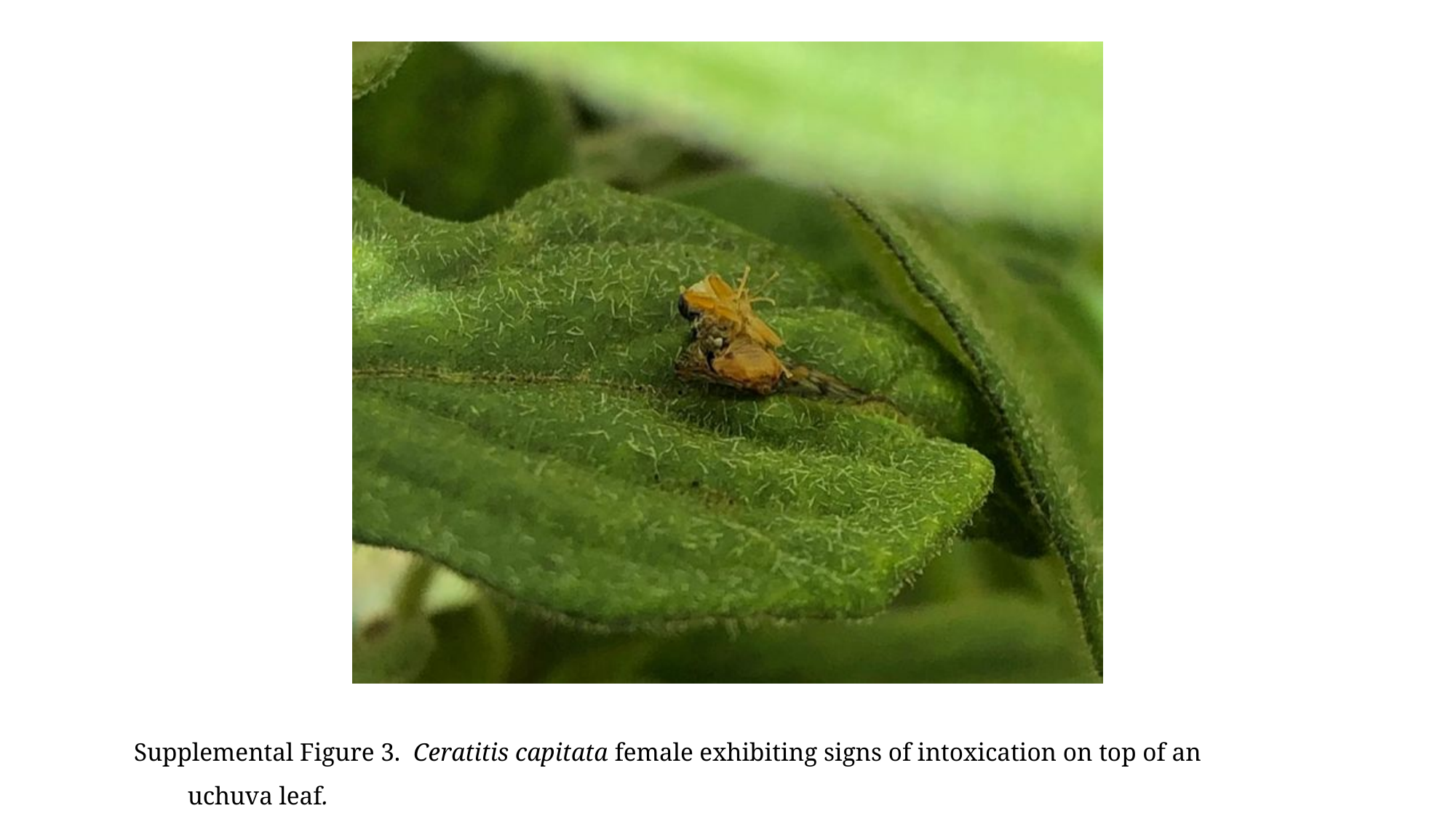

Supplemental Figure 3. Ceratitis capitata female exhibiting signs of intoxication on top of an uchuva leaf.
