# Supplementary material for: Physalis peruviana L. (Solanaceae) Is Not a Host of Ceratitis capitata (Diptera: Tephritidae): Evidence from Multi-Year Field and Laboratory Studies in Colombia
Source: Insects. 2019 Dec 4;10(12):434. doi: 10.3390/insects10120434 (PMC6956068; doi:10.3390/insects10120434)
Supplement: Supplementary file 1 [file insects-10-00434-s001.zip › Supplemental Table S1.docx]

**Supplemental Table S1.** Plant species sampled over a three-year period in the Departamento Norte de Santander (details on sampling routes are provided in Fig. 2) to identify *Ceratitis* *capitata* local hosts and in the case of positive findings, degree of infestation (number of larvae/kg fruit).

| **Fruit species** | **Local common name** | **Cultivar** | **Fruit fly species** | **Fruit sampled (Kg)** | **Number of fruit sampled** | **Number of infested fruit** | **Total no. of larvae** | **Infestation (Larvae/Kg of fruit)** |
| --- | --- | --- | --- | --- | --- | --- | --- | --- |
| *Acca sellowiana* (O. Berg) | Feijoa |  | Total | 57.0 | 1,713 | 68 | 151 | 2.7 |
|  |  |  | *Anastrepha fraterculus* complex | 3.4 | 112 | 39 | 62 | 18.2 |
|  |  |  | *Ceratitis capitata* | 3.4 | 87 | 29 | 89 | 26.6 |
| *Annona cherimola* Mill. | Chirimoya |  | NR | 5.2 | 21 | 0 | 0 | 0.0 |
| *Caesalpinia spinosa* (Molina) | Dividivi |  | NR | 0.4 | 56 | 0 | 0 | 0.0 |
| *Capsicum spp.* | Ají |  | Total | 14.0 | 569 | 36 | 42 | 3.0 |
|  |  |  | *C. capitata* | 1.1 | 117 | 36 | 42 | 38.2 |
| *Capsicum annuum* L. | Pimentón |  | NR | 7.9 | 149 | 0 | 0 | 0.0 |
| *Carica papaya* L. | Papaya |  | NR | 1.8 | 3 | 0 | 0 | 0.0 |
| *Chrysophyllum argentum* Jacq. | Caimas |  | NR | 0.1 | 272 | 0 | 0 | 0.0 |
| *Citrus × aurantifolia* (Christm.) | Limón |  | NR | 13.8 | 177 | 0 | 0 | 0.0 |
|  | Limón agrio |  | NR | 5.1 | 50 | 0 | 0 | 0.0 |
|  | Limonson |  | NR | 13.3 | 161 | 0 | 0 | 0.0 |
| *Citrus × aurantium* L. | Naranja agria |  | NR | 9.7 | 71 | 0 | 0 | 0.0 |
| *Citrus × limonia* Osbeck | Limón mandarino |  | NR | 10.2 | 156 | 0 | 0 | 0.0 |
| *Citrus reticulata* Blanco | Mandarina |  | Total | 29.1 | 411 | 2 | 18 | 0.6 |
|  |  |  | *Neosilba* sp. | 1.0 | 11 | 2 | 18 | 18.0 |
| *Citrus sinensis* (L.) | Naranja |  | Total | 143.1 | 1,330 | 7 | 19 | 0.1 |
|  |  |  | *Neosilba* sp | 2.7 | 34 | 6 | 14 | 5.2 |
|  |  |  | *C. capitata,* *Neosilba* sp. | 2.0 | 12 | 1 | 5 | 2.5 |
| *Citrus spp.* | Lima |  | NR | 8.5 | 62 | 0 | 0 | 0.0 |
| *Citrus paradisi* Macfad. | Toronja |  | NR | 15.2 | 18 | 0 | 0 | 0.0 |
| *Clusia multiflora* Kunth*.* | Gaque |  | NR | 0.2 | 20 | 0 | 0 | 0.0 |
| *Coffea arabica* L. | Café |  | Total | 42.4 | 19,756 | 556 | 528 | 12.5 |
|  |  |  | *A. fraterculus* complex | 0.9 | 529 | 18 | 20 | 22.2 |
|  |  |  | *C. capitata* | 6.8 | 2,839 | 509 | 476 | 70.2 |
|  |  |  | *Neosilba* sp. | 0.3 | 108 | 7 | 10 | 40.0 |
|  |  |  | *C. capitata, A. fraterculus* complex | 0.8 | 418 | 22 | 22 | 27.5 |
| *Cucumis sativus* L. | Pepino cohombro |  | NR | 1.7 | 8 | 0 | 0 | 0.0 |
| *Cucurbita pepo* L. | Zapallo |  | NR | 2.0 | 6 | 0 | 0 | 0.0 |
| *Cucurbita* spp. | Calabacín |  | NR | 2.3 | 4 | 0 | 0 | 0.0 |
| *Cyclanthera pedata* (L.) Schrad. | Pepino rellenar |  | NR | 0.5 | 5 | 0 | 0 | 0.0 |
| *Eriobotrya japonica* (Thunb.) | Níspero japonés |  | Total | 27.8 | 1,538 | 50 | 78 | 2.8 |
|  |  |  | *A. fraterculus* complex | 1.0 | 62 | 37 | 52 | 52.0 |
|  |  |  | *C. capitata* | 2.5 | 126 | 13 | 26 | 10.4 |
| *Erythrina edulis* Triana ex Micheli | Chachafruto |  | NR | 1.5 | 38 | 0 | 0 | 0.0 |
| *Eugenia* spp. | Eugenias |  | NR | 1.3 | 640 | 0 | 0 | 0.0 |
| *Eugenia stipitata* McVaugh. | Arazá |  | NR | 0.6 | 330 | 0 | 0 | 0.0 |
| *Ficus carica* L. | Breva |  | Total | 36.1 | 884 | 2 | 10 | 0.3 |
|  |  |  | *C. capitata, Neosilba* sp. | 1.0 | 25 | 2 | 10 | 10.0 |
| *Ficus indica* (L.) | Higo |  | NR | 6.0 | 162 | 0 | 0 | 0.0 |
| *Fragaria* spp. | Fresa |  | NR | 1.1 | 80 | 0 | 0 | 0.0 |
| *Juglans regia* L. | Nogal |  | Total | 8.5 | 216 | 92 | 247 | 29.1 |
|  |  |  | *Anastrepha manizaliensis* | 2.3 | 134 | 91 | 245 | 106.5 |
|  |  |  | *Lonchaea* sp. | 1.4 | 14 | 1 | 2 | 1.4 |
| *Malus domestica* Borkh. | Manzana ana |  | Total | 59.2 | 1,053 | 7 | 54 | 0.9 |
|  |  |  | *C. capitata* | 1.6 | 37 | 7 | 54 | 33.8 |
|  | Manzana | Criollo | NR | 4.6 | 83 | 0 | 0 | 0.0 |
| *Mangifera indica* L. | Mango |  | NR | 0.8 | 4 | 0 | 0 | 0.0 |
| *Morinda citrifolia* L. | Noni |  | NR | 2.3 | 26 | 0 | 0 | 0.0 |
| *Opuntia* spp*.* | Cactus |  | NR | 27.6 | 663 | 0 | 0 | 0.0 |
| *Passiflora edulis f. flavicarpa* O. Deg. | Maracuyá |  | NR | 1.0 | 9 | 0 | 0 | 0.0 |
| *Passiflora edulis f.edulis* Sims. | Gulupa |  | Total | 73.5 | 1,677 | 54 | 156 | 2.1 |
|  |  |  | *Dasiops gracilis* | 1.5 | 54 | 32 | 132 | 88.0 |
|  |  |  | *Neosilba* sp. | 0.6 | 28 | 22 | 24 | 40.0 |
| *Passiflora ligularis* Juss. | Granadilla |  | NR | 30.2 | 316 | 0 | 0 | 0.0 |
|  | Granadilla | Criollo | NR | 0.2 | 9 | 0 | 0 | 0.0 |
| *Passiflora quadrangularis* L. | Badea melocotón |  | NR | 6.0 | 4 | 0 | 0 | 0.0 |
| *Passiflora tripartita* (Juss.) | Curuba silvestre |  | NR | 0.4 | 20 | 0 | 0 | 0.0 |
|  | Curuba | Simarrona | NR | 0.2 | 8 | 0 | 0 | 0.0 |
| *Passiflora tripartita var. mollissima* (Kunth) | Curuba |  | Total | 74.1 | 1,029 | 5 | 11 | 0.1 |
|  |  |  | *Neosilba* sp. | 2.1 | 34 | 5 | 11 | 5.2 |
| *Persea americana* Mill. | Aguacate | Criollo | NR | 32.1 | 65 | 0 | 0 | 0.0 |
| *Physalis peruviana* L. | Uchuva comercial |  | NR | 630.0 | 92,503 | 0 | 0 | 0.0 |
| *Physalis peruviana* L. | Uchuva 'feral' |  | NR | 32.5 | 5,629 | 0 | 0 | 0.0 |
| *Prunus domestica* L. | Ciruela |  | NR | 44.3 | 1,662 | 0 | 0 | 0.0 |
|  | Ciruela | Amarilla | NR | 0.5 | 15 | 0 | 0 | 0.0 |
|  | Ciruela | Horvin | NR | 4.1 | 194 | 0 | 0 | 0.0 |
|  | Ciruela | Roja | NR | 7.1 | 269 | 0 | 0 | 0.0 |
| *Prunus persica* (L.) | Durazno |  | Total | 47.0 | 530 | 42 | 209 | 4.4 |
|  |  |  | *C. capitata* | 5.6 | 80 | 29 | 208 | 37.1 |
|  |  |  | *Neosilba* sp. | 1.5 | 14 | 13 | 1 | 0.7 |
| *Prunus persica* (L.) | Durazno | Jarillo | Total | 22.2 | 264 | 14 | 16 | 0.7 |
|  | Durazno | Jarillo | *C. capitata* | 1.5 | 16 | 14 | 16 | 10.7 |
|  | Durazno | Gran jarillo | NR | 10.1 | 165 | 0 | 0 | 0.0 |
| *Psidium guajava* L. | Guayaba | Agria | Total | 20.7 | 1,260 | 135 | 190 | 9.2 |
|  | Guayaba |  | *Anastrepha striata* | 2.3 | 142 | 70 | 98 | 42.6 |
|  | Guayaba |  | *C. capitata* | 2.0 | 108 | 65 | 92 | 46.0 |
|  | Guayaba | Blanca | NR | 2.5 | 83 | 0 | 0 | 0.0 |
|  | Guayaba | Criolla | Total | 63.2 | 1,845 | 141 | 521 | 8.2 |
|  | Guayaba |  | *A. striata* | 10.0 | 256 | 124 | 470 | 47.2 |
|  | Guayaba |  | *C. capitata* | 1.9 | 26 | 17 | 51 | 26.8 |
|  | Guayaba silvestre |  | NR | 1.2 | 131 | 0 | 0 | 0.0 |
| *Punica granatum* L. | Granada |  | NR | 1.5 | 12 | 0 | 0 | 0.0 |
| *Quararibea cordata* (Bonpland) | Zapote |  | NR | 0.6 | 4 | 0 | 0 | 0.0 |
| *Rubus glaucus* Benth | Mora |  | NR | 0.1 | 24 | 0 | 0 | 0.0 |
| *Solanum lycopersicum var. Cerasiforme* | Tomate | Cherry | NR | 2.7 | 703 | 0 | 0 | 0.0 |
| *Solanum melongena* L*.* | Berenjena |  | NR | 3.9 | 8 | 0 | 0 | 0.0 |
| *Solanum pseudocapsicum L.* | Mirto |  | NR | 3.6 | 1,325 | 0 | 0 | 0.0 |
| *Solanum quitoense Lam.* | Lulo |  | NR | 47.5 | 564 | 0 | 0 | 0.0 |
| *Solanum betaceum Cav.* | Tomate de árbol |  | NR | 230.2 | 2,585 | 0 | 0 | 0.0 |
| *Solanum lycopersicum L.* | Tomate | Chonto | *Lonchaea* sp. | 0.6 | 32 | 1 | 16 | 29.1 |
|  | Tomate de mesa |  | NR | 11.9 | 171 | 0 | 0 | 0.0 |
| *Vaccinium floribundum Kunth.* | Cucano |  | NR | 1.2 | 700 | 0 | 0 | 0.0 |
| *Vasconcellea pubescens A.DC.* | Papayuela |  | NR | 15.2 | 133 | 0 | 0 | 0.0 |
| *Vasconcellea pubescens A.DC.* | Papayuela | Zapira | NR | 7.8 | 59 | 0 | 0 | 0.0 |

NR = None recovered
