# Supplementary material for: Physalis peruviana L. (Solanaceae) Is Not a Host of Ceratitis capitata (Diptera: Tephritidae): Evidence from Multi-Year Field and Laboratory Studies in Colombia
Source: Insects. 2019 Dec 4;10(12):434. doi: 10.3390/insects10120434 (PMC6956068; doi:10.3390/insects10120434)
Supplement: Supplementary file 1 [file insects-10-00434-s001.zip › Supplemental Table S2.docx]

**Supplemental Table S2.** *Physalis* *peruviana* export statistics from Colombia to 36 countries, including the US from 2015 to 2018. Source: Sistema de Información Sanitaria para Importación y Exportación de Productos Agrícolas y Pecuarios-Instituto Colombiano Agropecuario (SISPAP- 2019).

| Country | Amount (Kg) | | | | | |
| --- | --- | --- | --- | --- | --- | --- |
|  | 2015 | | 2016 | 2017 | 2018 | Total |
| Germany | | 109,578 | 171,830 | 82,425 | 53,539 | 417,372 |
| Saudi Arabia | | 54 | 116 | 277 | 162 | 609 |
| Aruba | | 103 | 42 |  |  | 144 |
| Bahrein | | 14 | 198 | 215 | 34,856 | 35,283 |
| Belgium | | 730,904 | 76,774 | 165,744 | 10,793 | 984,214 |
| Brazil | | 107,907 | 119,024 | 148,774 | 172,646 | 548,350 |
| Canada | | 92,656 | 107,352 | 164,364 | 238,943 | 603,317 |
| Curazao | | 288 |  |  |  | 288 |
| People's Republic of China (PRC) | |  | 100 | 3 |  | 103 |
| United Arab Emirates | | 4,288 | 8,139 | 9,695 | 14,191 | 36,313 |
| Spain | | 23,792 | 56,541 | 44,925 | 51,410 | 176,669 |
| United States of America (USA) | | 77,277 | 234,849 | 379,595 | 676,702 | 1’368,423 |
| France | | 10,890 | 60,010 | 96,548 | 48,260 | 215,709 |
| Greece | |  |  | 251 | 78 | 329 |
| Guadalupe | |  | 297 |  |  | 297 |
| Guatemala | | 84 | 316 | 395 | 145 | 940 |
| Holland (Netherlands) | | 4’348,833 | 4’288,688 | 4’893,463 | 5’604,047 | 19’135,030 |
| Hong Kong | | 231 |  |  | 3 | 234 |
| Indonesia | |  | 75 |  |  | 75 |
| England | | 2,298 | 1,995 | 11,003 | 2,663 | 17,959 |
| Ireland | | 1,095 |  | 113 |  | 1,208 |
| Italy | | 1,010 | 2,418 | 10,269 | 7,040 | 20,736 |
| Jordan | |  | 101 |  |  | 101 |
| Kuwait | |  | 155 | 135 | 217 | 506 |
| Lebanon | | 7 |  |  |  | 7 |
| Malaysia | | 258 | 252 | 481 | 1,837 | 2,828 |
| Norway | | 3 |  |  |  | 3 |
| Panama | | 8,443 | 5,472 | 8,153 | 1,412 | 23,480 |
| Poland | |  | 5 |  | 18 | 23 |
| Portugal | | 4,151 | 207 | 2,615 | 13 | 6,987 |
| Qatar | | 466 |  |  |  | 466 |
| UK | | 1,482 | 3,958 | 1,143 | 928 | 7,511 |
| Russia | | 1,094 | 3,123 | 1,780 | 4,886 | 10,882 |
| Singapore | | 32 | 19 |  | 132 | 184 |
| Switzerland | | 7,369 | 11,566 | 4,230 | 7,591 | 30,755 |
| Ukraine | |  |  |  | 134 | 134 |
| Total per year | | 5’534,607 | 5’153,621 | 6’026,594 | 6’932,647 | 23’647,469 |
